# Cormic Index Profile of Children with Sickle Cell Anaemia in Lagos, Nigeria

**DOI:** 10.1155/2014/312302

**Published:** 2014-04-17

**Authors:** Samuel Olufemi Akodu, Olisamedua Fidelis Njokanma, Omolara Adeolu Kehinde

**Affiliations:** Department of Paediatrics, Lagos State University Teaching Hospital, P.O. Box 11950, Ikeja, Lagos 100001, Nigeria

## Abstract

*Background*. Sickle cell disorders are known to have a negative effect on linear growth. This could potentially affect proportional growth and, hence, Cormic Index. *Objective*. To determine the Cormic Index in the sickle cell anaemia population in Lagos. *Methodology*. A consecutive sample of 100 children with haemoglobin genotype SS, aged eight months to 15 years, and 100 age and sex matched controls (haemoglobin genotype AA) was studied. Sitting height (upper segment) and full length or height were measured. Sitting height was then expressed as a percentage of full length/height (Cormic Index). *Results*. The mean Cormic Index decreased with age among primary subjects (SS) and AA controls. The overall mean Cormic Index among primary subjects was comparable to that of controls (55.0 ± 4.6% versus 54.5 ± 5.2%; 54.8 ± 4.5% versus 53.6 ± 4.9%) in boys and girls, respectively. In comparison with AA controls, female children with sickle cell anaemia who were older than 10 years had a significantly lower mean Cormic Index. *Conclusion*. There was a significant negative relationship between Cormic Index and height in subjects and controls irrespective of gender. Similarly, a significant negative correlation existed between age, sitting height, subischial leg length, weight, and Cormic Index in both subjects and controls.

## 1. Introduction

Sickle cell anaemia is a group of genetic disorders most commonly seen in man and it is found in people of African descent but it is also seen in people of other ethnic groups [1]. It has been shown that, as a group, children with sickle cell anaemia have poor growth [2, 3]. Anthropometry is the principal method of assessing growth and height/length-for-age is the most useful linear measurement that gives an indication of past nutrition [4].

The Cormic Index expresses sitting height as a proportion of full height. It is a measure of the relative length of trunk and lower limb and it varies between individuals and groups [5]. It is the most common bivariate index of shape [5]. The Cormic Index is most often used to correct for variability in body shape when Body Mass Index (BMI) is used to compare the nutritional status in or between different populations [6]. For example, individuals who are very tall may be wrongly classified as being underweight despite having normal body weight for their culture. Similarly, in muscular people BMI may be more likely to indicate overweight, as lean body mass tissue is denser than fat tissue. The BMI can be modified using Cormic Index to help correct for this variability in body shape. Used as such, it adjusts for population differences in phenotype that may impact BMI. It is important to recognize that this type of adjustment mainly has been applied to adults and not to children or adolescents [7].

It has been evident that there is variability in the growth of spinal length compared with limb length during the prepubertal period and during adolescence. Previous studies have shown that increase in sitting height is relatively faster than leg length in later childhood [8, 9]. The negative effect of sickle cell anaemia on spinal growth and, hence, Cormic Index would therefore be expected to be more obvious in older children during the period of expected rapid growth. Shorter spinal length relative to the height increases the Cormic Index.

To the best of the authors' knowledge, there is no prior report of the Cormic Index among children with sickle cell anaemia in Nigeria or elsewhere. Studies have been conducted among African [10], Australian aborigines [11], and Asian [12] population without specific reference to haemoglobin genotype. However, the direct effect of sickle cell anaemia on linear growth may limit the application of findings in the general population to affected children.

The appraisal of the Cormic Index among children with sickle cell anaemia is therefore of clinical and scientific interest. Therefore, the main objective of the present report was to determine the Cormic Index among children with sickle cell anaemia.

## 2. Materials and Methods

The cross-sectional study was conducted between October and December 2009 among children with sickle cell anaemia attending the sickle cell disease clinic of the Department of Paediatrics of Lagos State University Teaching Hospital, Ikeja, in Southwest Nigeria. The hospital is an urban tertiary health centre in Lagos State, Western Nigeria. It is a major referral center serving the whole state, which is a major point of entry into Nigeria from different parts of the world and is the economic nerve centre of Nigeria.

Approval for the study was obtained from the Ethics Committee of Lagos State University Teaching Hospital and written informed consent was obtained from each parent. Consecutive patients with sickle cell anaemia who came for routine follow-up clinic that gave consent and met the study criteria were eligible for enrollment in the study. Healthy controls were children with haemoglobin genotype “AA” from the general outpatient and follow-up clinics and healthy children attending other specialist clinics like the Paediatric Dermatology Clinic. Controls were matched with primary subjects for age and sex. Two hundred children were studied—one hundred each with haemoglobin genotypes SS and AA. In order to have fairly equal representation of ages, the subjects stratified as follows: <2 years, >2 to 5 years, >5 to 10 years, and >10 to 15 years.

### 2.1. Inclusion Criteria

1. Age six months to fifteen years.
2. Confirmed HbSS by electrophoresis.
3. Signed, informed consent of the caregiver.
4. Subjects who were in steady state, that is, absence of any crisis in the preceding four weeks, no recent drop in the haemoglobin level, and absence of any symptoms or signs attributable to acute illness [13].
5. Children who were not taking medications known to affect growth, for example, steroids.

### 2.2. Exclusion Criteria

1. Children with congenital cardiac abnormality, chronic renal disease, or abnormal chest wall deformity or chronic respiratory disorder.
2. Refusal of consent.
3. Children with history of cerebrovascular accident.
4. Sickle cell anaemia patients with history of long-term transfusion therapy.

The inclusion and exclusion criteria for the controls were the same as for subjects except that the haemoglobin genotype was AA.

### 2.3. Measurement of Height

Children two years of age and older had their heights measured using a stadiometer while the length of those below two years was measured using an infantometer.

### 2.4. Measurement of Weight

Subjects' weights were measured barefooted and wearing light clothing. Weight measurements were taken on a Seca 761 series mechanical floor scale to the nearest 0.1 Kg.

### 2.5. Measurement of Sitting Height

Sitting height was measured using a sitting height table. Sitting height was measured from the vertex of the head to the seated buttocks. The subject's head was positioned in the Frankfort horizontal plane, the shoulders relaxed, the back straight, and the head plate was brought into firm contact with the vertex [14].

The various linear measurements were taken three times and the mean was recorded. Subjects were measured wearing light clothing and shoes were removed. The measurements were taken following the standard techniques. The measurements were carried out by the researchers.

### 2.6. Derivation of Subischial Leg Length

It is expressed as the difference between height/length and sitting height.

### 2.7. Derivation of Body Mass Index

The body mass index was expressed as weight in Kg/height in metre² (Kg/m²).

### 2.8. Derivation of Cormic Index

It is expressed as

(1) $$\begin{array}{c} \left(\frac{\text{Sitting}\;\text{height}}{\text{height}}\right)\times 100. \end{array}$$

Social classification was done using the scheme proposed by Oyedeji [15] in which subjects are grouped into five classes (I–V) based on the occupation and educational attainments of both parents. Analysis was done using Statistical Package for Social Science (SPSS) version 17.0. Comparison of mean values was done using Student's *t*-test and *P* < 0.05 is considered significant. The Pearson correlation coefficient (*r*) was attempted for understanding the overall relationship of anthropometric variables and age with Cormic Index.

In order to standardize the calculated BMI, the model developed by Norgan [16] was modified and applied. In the original model, standardized BMI was obtained using the formula BMI_std_ = BMI_52.  0_ + (BMI_0_ − BMI_1_), where

 BMI_std_ is standardized BMI,
 BMI_52.  0_ is estimated BMI at Cormic Index of 54.9% (the mean Cormic Index for the European population),
 BMI_0_ is actual (observed) BMI,
 BMI_1_ is estimated BMI at actual (observed) Cormic Index.
 Note: Cormic Index should be expressed as a percentage.

Modification to the Norgan model was done by substituting the mean Cormic Index of the European population with that for the haemoglobin AA population in the current study.

## 3. Results

### 3.1. Cormic Index of Study Subjects

The Cormic Index of the SS subjects ranged from 44.5% to 68.2% while that for AA controls ranged from 45.9% to 65.0%. The mean Cormic Index of the SS group of 54.1 (±5.1)% was not statistically different from 54.9 (±4.5)% in the AA-control group (*t*-value = 1.240, *P* = 0.216). The overall mean Cormic Index was 54.5 (±4.8)%. The mean Cormic Index of the study subjects according to age, gender, and socioeconomic strata was shown in Table 1. Mean Cormic Index decreased with age irrespective of gender or haemoglobin genotype. In each age group, the value observed in males with genotype SS was comparable to that of their AA counterparts. This was also the pattern in females except in the oldest age group in which AA controls had a significantly higher index. Within the haemoglobin “SS” group, the mean Cormic Index values of males were comparable to those of females across all age groups. The same was true of “AA” subjects across all age groups.

The mean Cormic Index was significantly higher in sickle cell anaemia subjects of the upper socioeconomic class than in those of the other classes (*P* = 0.047). On the other hand, the difference between mean values for upper class and lower class controls was not significant (*P* = 0.63).

### 3.2. Correlation between Cormic Index and Height

Table 2 shows the results of correlation analysis between Cormic Index and height of study subjects with and without sickle cell anaemia. Overall, the Cormic Index had strong negative correlations with height (*r* = −0.850, −0.860, in subjects and controls, resp.). The pattern of negative correlation was observed in both sexes and in all age groups but the coefficients were not consistently significant. Significant positive correlations were detected between sitting height and subischial leg length (*r* = 0.895, 0.925: *P* = 0.000 each) in subjects and controls, respectively.

### 3.3. Correlation between Cormic Index and Other Anthropometrics and Age

Anthropometric variables tested were weight, BMI, sitting height, and subischial leg length. The Pearson correlation coefficient (*r*) for understanding the overall relationship of anthropometric variables and age with Cormic Index is shown in Table 3. Examination on the Pearson correlation coefficient revealed a significant (*P* < 0.05) negative correlation between age, sitting height, subischial leg length, weight, and Cormic Index in both subjects and controls. Also, a weak correlation was observed between BMI and Cormic Index among subjects with HbSS and controls. However, it was in subjects with sickle cell anaemia that the correlation coefficient was significant (*P* = 0.000). Table 3 also shows that the correlation between Cormic Indices and subischial leg length is higher in both subjects with sickle cell anaemia and controls.

### 3.4. Regression of BMI and Cormic Index

A simple regression equation was derived from the relationship between BMI as dependent variable and Cormic Index as independent variable (Table 4). Separate regression equations for the sexes were derived for BMI on the Cormic Index. Testing by covariance analysis showed that the slopes and intercepts were not significantly different. Therefore, the sexes were combined and a single equation was used to calculate expected BMI values.

Standardized BMI using the modified Norgan model was obtained as follows:

(2) $$\begin{array}{c} \text{BM}{\text{I}}_{\text{std}}=\text{BM}{\text{I}}_{54.9}+\left(\text{BM}{\text{I}}_{0}-\text{BM}{\text{I}}_{1}\right). \end{array}$$

Using 54.9 (the mean Cormic Index of haemoglobin AA controls) in the regression equation for estimating BMI yielded a value of 14.5.

Thus, the final model is

(3) $$\begin{array}{c} \text{BM}{\text{I}}_{\text{std}}=14.5+\left(\text{BM}{\text{I}}_{0}-\text{BM}{\text{I}}_{1}\right). \end{array}$$

*z*-scores were generated for observed BMI as well as the standardized BMI. On the basis of the *z*-scores, study subjects were then categorized into “thin,” “normal,” or “overweight.” Table 5 shows the prevalence of thinness and overweight using both the actual (observed) and the standardized BMI values. Table 5 shows that, altogether, sixty subjects were classified as thin on the basis of *z*-scores of observed BMI. Of these 60, six were identified as thin using *z*-scores of standardized BMI. Also, five subjects were adjudged overweight using observed BMI: four of these five subjects were identified as overweight using standardized BMI.

## 4. Discussion

Cormic Indices were comparable between male and female children with sickle cell anaemia. Unfortunately, there are no local or international figures for comparison. However, mean Cormic Index decreased progressively with age both in primary subjects and in controls. This is consistent with the expected physiologic trend in which the lower limbs grow relatively faster than the trunk in early childhood.

Specifically, comparison of mean Cormic Index between HbSS subjects and controls older than 10 years showed that controls have significantly higher mean Cormic Index values than sickle cell anaemia subjects irrespective of gender. Interestingly, the significant difference was not recorded in males following gender stratification. The explanation for the different pattern in males and females is not clear. This significantly lower mean value among females with sickle cell anaemia older than 10 years translates to relatively shorter trunks which may be explained by narrowing of intervertebral discs as a result of repeated vasoocclusion [17, 18]. Added to this explanation is the fact that increase in sitting height is relatively faster than leg length in later childhood [8, 19]. The negative effect of sickle cell anaemia on spinal growth would therefore be expected to be more obvious in older children during the period of expected rapid growth.

Thus, the finding of lower Cormic Index in older subjects might be expected but the limitation of the observation to girls is not readily explained. It is plausible that the observation represents a relatively minor trend that has been exaggerated by peculiar circumstances of the female subjects in the current study. Only further studies, possibly with much larger subgroups, can elucidate the situation. A plausible explanation could be that there was a fortuitous concentration of female sickle cell anaemia with relatively less severe affection of height.

Significant negative correlations were detected between Cormic Index and height (*r* = −0.868, −0.855) in boys and girls, respectively. This is purely an arithmetical relationship: height is the denominator in the Cormic Index. Therefore, the ratio should increase as the denominator reduces and vice versa.

It was also observed that strong negative correlations existed between Cormic Index and age (0.752, 0.744). Similar observations have been reported in a study of healthy Bengalee children aged six years to 12 years [5]. Both the sitting height and height are linear measurements which increase physiologically in the same direction with age. Arithmetically, this ratio could be reduced if the sitting height is relatively short. Several previous studies have shown that increase in sitting height is faster than leg length in later childhood [20, 21]. A disease like sickle cell anaemia that affects growth is therefore more likely to adversely affect sitting height in later childhood.

From the result of this study there is significant positive correlation when sitting height was compared to subischial height. This study has also demonstrated that Cormic Index has a direct relationship with sitting height and subischial leg length. That is to say, it is the size of the trunk that mainly determines the body Cormic Index and not subischial leg length.

A positive correlation exists between Cormic Index and BMI in subjects with sickle cell anaemia and controls, although this correlation is relatively weak (<0.4). The low *r*-values indicate that the Cormic Index is a minor determinant of BMI. This corroborates a study of Nigerians aged between 15 and 56 years in whom weak positive correlation between Cormic Index and BMI was observed [22].

BMI is known to vary with age and body shape. The cut-off used for BMI classification is the same in both children with and children without sickle cell anaemia. There is a marked difference between body shape of children with sickle cell anaemia and that without sickle cell anaemia. In order to account for changes in this documented body shape, the Cormic Index was standardized to compare the BMI of different haemoglobin genotype populations to prevent or reduce the overestimation of prevalence of BMI abnormalities. Upon standardization, the current study showed a 90% reduction in the proportion of subjects otherwise classified as thin. The effect of the standardization was far less felt at the upper end of the BMI spectrum. Indeed, there was only a 17% reduction in the number of subjects classified as overweight. It is thus attractive to argue that the standardization will be more relevant when the objective was to determine proportion of thinness among subjects with sickle cell anaemia.

The extent to which the standardization in the current study applies across races or ethnic groups can only be confirmed by further study. Also, it is plausible that severity of illness may influence the interrelationships between Cormic Index and BMI measurements. Thus, it may be argued that regions with milder or more severe disease expressions may require developing their own standardization models.

In conclusion, the mean Cormic Index decreased with age in both subjects and controls. The mean Cormic Index for children with sickle cell anaemia is comparable with that of HbAA controls except in females older than 10 years that the mean Cormic Index was significantly higher among AA controls than their SS counterparts. The correlation analysis between Cormic Index and height is strongly negative. This may be of great importance for the field of anthropology and forensic medicine. Adjusting for body proportions in classifying BMI drastically reduced the number of sickle cell anaemia subjects who would have been categorized as thin.

## Figures and Tables

**Table 1:** Mean Cormic Index distribution of study subjects according to age and gender.

Variable	SS	AA	*t*-value	*P* value
	Mean (SD)	Mean (SD)		
Age group				
≤2 yrs				
Males	60.3 (2.6)	60.1 (1.8)	−0.228	0.822
Females	59.5 (2.8)	60.8 (2.6)	1.192	0.245
Males and females	59.9 (2.7)	60.5 (2.2)	0.783	0.437
>2 yrs–5 yrs				
Males	54.5 (2.3)	56.6 (3.3)	1.914	0.068
Females	55.0 (2.3)	54.8 (1.5)	−0.267	0.792
Males and females	54.7 (2.2)	55.7 (2.7)	1.408	0.165
>5 yrs–10 yrs				
Males	52.1 (5.6)	51.5 (1.2)	−0.385	0.704
Females	51.4 (1.6)	51.6 (2.2)	0.337	0.739
Males and females	51.8 (4.0)	51.6 (1.7)	−0.208	0.836
>10 yrs–15 yrs				
Males	50.4 (3.6)	51.1 (3.5)	0.475	0.640
Females	48.1 (2.2)	51.5 (2.9)	3.181	0.004
Males and females	49.3 (3.1)	51.3 (3.2)	2.213	0.032
Socioeconomic strata				
Upper	55.15 (5.15)	55.02 (4.89)	0.128	0.899
Other	53.10 (4.70)	54.57 (4.18)	1.612	0.110

SD: standard deviation.

**Table 2:** Correlation analysis between height and Cormic Index in study subjects.

Age group	Correlation coefficient (*r*)		*P* value	
	AA	SS	AA	SS
≤2 yrs				
Males	−0.679	−0.411	0.011	0.164
Females	−0.786	−0.604	0.001	0.029
Males and females	−0.708	−0.465	0.000	0.017
>2 yrs–5 yrs				
Males	−0.887	−0.680	0.000	0.011
Females	−0.571	−0.861	0.042	0.000
Males and females	−0.827	−0.769	0.000	0.000
>5 yrs–10 yrs				
Males	−0.460	−0.641	0.133	0.025
Females	−0.594	−0.235	0.042	0.462
Males and females	−0.552	−0.569	0.005	0.004
>10 yrs–15 yrs				
Males	−0.453	−0.644	0.139	0.024
Females	−0.299	−0.047	0.345	0.885
Males and females	−0.381	−0.495	0.066	0.014

**Table 3:** The Pearson correlation of Cormic Index with other anthropometrics and age.

Characteristics	Correlation coefficient (*r*)		*P* value	
	AA	SS	AA	SS
Age	−0.752	−0.744	0.000	0.000
BMI	0.120	0.386	0.241	0.000
Subischial leg length	−0.922	−0.919	0.000	0.000
Sitting height	−0.728	−0.670	0.000	0.000
Weight	−0.565	−0.700	0.000	0.000

**Table 4:** Regression of BMI on Cormic Index of subjects and controls.

Independent variables	Equation	*R* ^2^	SEE (cm)
Sickle cell anaemia subjects			
Males	3.73 + 0.21 CI	0.162	2.448
Females	6.10 + 0.15 CI	0.126	1.951
Males and females	4.60 + 0.18 CI	0.149	2.215
Controls			
Males	6.52 + 0.16 CI	0.063	2.901
Females	15.26 + 0.02 CI	0.000	3.961
Males and females	10.84 + 0.09 CI	0.014	3.459

CI: Cormic Index.

Note: Cormic Index should be expressed as a percentage.

**Table 5:** The effect of adjusting the BMI of subjects with sickle cell anaemia for a mean Cormic Index of 54.9%.

Age group	Thinness		Overweight	
	Actual	Adjusted	Actual	Adjusted
≤2 yrs				
Males	8 (61.5)	4 (30.8)	1 (7.7)	0 (0.0)
Females	4 (30.8)	1 (7.7)	1 (7.7)	1 (7.7)
Males and females	12 (46.2)	5 (19.2)	2 (7.7)	1 (7.7)
>2 yrs–5 yrs				
Males	7 (53.8)	0 (0.0)	1 (7.7)	0 (0.0)
Females	7 (53.8)	0 (0.0)	0 (0.0)	0 (0.0)
Males and females	14 (53.8)	0 (0.0)	1 (3.9)	0 (0.0)
>5 yrs–10 yrs				
Males	5 (41.7)	0 (0.0)	1 (8.3)	1 (8.3)
Females	8 (66.7)	1 (8.3)	0 (0.0)	0 (0.0)
Males and females	13 (54.2)	1 (4.2)	1 (8.3)	1 (8.3)
>10 yrs–15 yrs				
Males	9 (75.0)	0 (0.0)	1 (8.3)	2 (16.7)
Females	12 (100)	0 (0.0)	0 (0.0)	0 (0.0)
Males and females	21 (87.5)	0 (0.0)	1 (4.2)	1 (4.2)
All				
Males	29 (58.0)	4 (8.0)	4 (8.0)	3 (6.0)
Females	31 (82.0)	2 (4.0)	1 (2.0)	1 (2.0)
Males and females	60 (60.0)	6 (6.0)	5 (5.0)	4 (4.0)
